# Supplementary material for: Pyruvate Dehydrogenase Kinase 1 inhibition mediated oxidative phosphorylation enhancement in cartilage promotes osteoarthritis progression
Source: BMC Musculoskelet Disord. 2023 Jul 20;24:597. doi: 10.1186/s12891-023-06585-6 (PMC10357736; doi:10.1186/s12891-023-06585-6)
Supplement: Supplementary file 1 — Additional file 1. [file 12891_2023_6585_MOESM1_ESM.zip › MF.Gsea.1653627316242/GOMF_CARBOXY_LYASE_ACTIVITY.html]

Details for gene set GOMF\_CARBOXY\_LYASE\_ACTIVITY[GSEA]

|  || Dataset | 55457\_collapsed\_to\_symbols.cls.cls #OA\_versus\_normal.cls.cls #OA\_versus\_normal\_repos |
| Phenotype | cls.cls#OA\_versus\_normal\_repos |
| Upregulated in class | OA |
| GeneSet | GOMF\_CARBOXY\_LYASE\_ACTIVITY |
| Enrichment Score (ES) | 0.407264 |
| Normalized Enrichment Score (NES) | 1.4410585 |
| Nominal p-value | 0.054187194 |
| FDR q-value | 0.2752333 |
| FWER p-Value | 1.0 |
Table: GSEA Results Summary

  

Fig 1: Enrichment plot: GOMF\_CARBOXY\_LYASE\_ACTIVITY      
 Profile of the Running ES Score & Positions of GeneSet Members on the Rank Ordered List

  

| SYMBOL | TITLE | RANK IN GENE LIST | RANK METRIC SCORE | RUNNING ES | CORE ENRICHMENT || 1 | GOT1 | glutamic-oxaloacetic transaminase 1 [Source:HGNC Symbol;Acc:HGNC:4432] | 59 | 0.974 | 0.0955 | Yes |
| 2 | UXS1 | UDP-glucuronate decarboxylase 1 [Source:HGNC Symbol;Acc:HGNC:17729] | 91 | 0.926 | 0.1882 | Yes |
| 3 | GGCX | gamma-glutamyl carboxylase [Source:HGNC Symbol;Acc:HGNC:4247] | 331 | 0.734 | 0.2449 | Yes |
| 4 | PCK2 | "phosphoenolpyruvate carboxykinase 2, mitochondrial [Source:HGNC Symbol;Acc:HGNC:8725]" | 759 | 0.579 | 0.2712 | Yes |
| 5 | UROD | uroporphyrinogen decarboxylase [Source:HGNC Symbol;Acc:HGNC:12591] | 905 | 0.541 | 0.3154 | Yes |
| 6 | DDT | D-dopachrome tautomerase [Source:HGNC Symbol;Acc:HGNC:2732] | 1094 | 0.498 | 0.3519 | Yes |
| 7 | ME1 | malic enzyme 1 [Source:HGNC Symbol;Acc:HGNC:6983] | 1746 | 0.392 | 0.3416 | Yes |
| 8 | ME2 | malic enzyme 2 [Source:HGNC Symbol;Acc:HGNC:6984] | 1870 | 0.377 | 0.3708 | Yes |
| 9 | PPCDC | phosphopantothenoylcysteine decarboxylase [Source:HGNC Symbol;Acc:HGNC:28107] | 2251 | 0.332 | 0.3753 | Yes |
| 10 | GAD1 | glutamate decarboxylase 1 [Source:HGNC Symbol;Acc:HGNC:4092] | 2534 | 0.299 | 0.3842 | Yes |
| 11 | AZIN1 | antizyme inhibitor 1 [Source:HGNC Symbol;Acc:HGNC:16432] | 2786 | 0.271 | 0.3925 | Yes |
| 12 | UMPS | uridine monophosphate synthetase [Source:HGNC Symbol;Acc:HGNC:12563] | 2936 | 0.257 | 0.4073 | Yes |
| 13 | ME3 | malic enzyme 3 [Source:HGNC Symbol;Acc:HGNC:6985] | 4613 | 0.104 | 0.2877 | No |
| 14 | MLYCD | malonyl-CoA decarboxylase [Source:HGNC Symbol;Acc:HGNC:7150] | 4852 | 0.083 | 0.2778 | No |
| 15 | BCKDHA | branched chain keto acid dehydrogenase E1 subunit alpha [Source:HGNC Symbol;Acc:HGNC:986] | 5671 | 0.018 | 0.2161 | No |
| 16 | ODC1 | ornithine decarboxylase 1 [Source:HGNC Symbol;Acc:HGNC:8109] | 6153 | -0.016 | 0.1804 | No |
| 17 | HDC | histidine decarboxylase [Source:HGNC Symbol;Acc:HGNC:4855] | 6599 | -0.050 | 0.1509 | No |
| 18 | PDXDC1 | pyridoxal dependent decarboxylase domain containing 1 [Source:HGNC Symbol;Acc:HGNC:28995] | 7373 | -0.108 | 0.1020 | No |
| 19 | ECHDC1 | ethylmalonyl-CoA decarboxylase 1 [Source:HGNC Symbol;Acc:HGNC:21489] | 7532 | -0.121 | 0.1021 | No |
| 20 | DDC | dopa decarboxylase [Source:HGNC Symbol;Acc:HGNC:2719] | 8605 | -0.202 | 0.0396 | No |
| 21 | PCK1 | phosphoenolpyruvate carboxykinase 1 [Source:HGNC Symbol;Acc:HGNC:8724] | 9100 | -0.239 | 0.0258 | No |
| 22 | GAD2 | glutamate decarboxylase 2 [Source:HGNC Symbol;Acc:HGNC:4093] | 9249 | -0.252 | 0.0401 | No |
| 23 | PAICS | phosphoribosylaminoimidazole carboxylase and phosphoribosylaminoimidazolesuccinocarboxamide synthase [Source:HGNC Symbol;Acc:HGNC:8587] | 9475 | -0.272 | 0.0506 | No |
| 24 | AMD1 | adenosylmethionine decarboxylase 1 [Source:HGNC Symbol;Acc:HGNC:457] | 10415 | -0.363 | 0.0149 | No |
| 25 | MVD | mevalonate diphosphate decarboxylase [Source:HGNC Symbol;Acc:HGNC:7529] | 10934 | -0.423 | 0.0181 | No |
| 26 | CSAD | cysteine sulfinic acid decarboxylase [Source:HGNC Symbol;Acc:HGNC:18966] | 11649 | -0.525 | 0.0165 | No |
| 27 | PISD | phosphatidylserine decarboxylase [Source:HGNC Symbol;Acc:HGNC:8999] | 12581 | -0.785 | 0.0248 | No |
Table: GSEA details [plain text format]

  

Fig 2: GOMF\_CARBOXY\_LYASE\_ACTIVITY      
 Blue-Pink O' Gram in the Space of the Analyzed GeneSet

  

Fig 3: GOMF\_CARBOXY\_LYASE\_ACTIVITY: Random ES distribution      
 Gene set null distribution of ES for **GOMF\_CARBOXY\_LYASE\_ACTIVITY**
